# Crystal structure of [2-({[2-(dimethylamino-κ*N*)ethyl]imino-κ*N*}methyl)phenolato-κ*O*](1,10-phen­anthroline-κ^2^ *N*,*N*′)copper(II) perchlorate

**DOI:** 10.1107/S2056989023001767

**Published:** 2023-03-07

**Authors:** Anjaneyulu Mamindla, Manikandan Varadhan, Marappan Velusamy, Venkatasubramanian Ulaganathan, Venugopal Rajendiran

**Affiliations:** a Department of Chemistry, School of Basic and Applied Sciences, Central University of Tamil Nadu, Thiruvarur - 610 005, India; b Department of Chemistry, North Eastern Hill University, Shillong - 793 022, India; c School of Chemical & Biotechnology, SASTRA Deemed University, Thanjavur - 613 401, Tamil Nadu, India; Universidad de Los Andes Mérida, Venezuela

**Keywords:** mixed ligand copper(II) complexes, anti­cancer agents, crystal structure, square-pyramidal distorted trigonal–bipyramidal (SPDTBP) geometry

## Abstract

The title compound, [Cu(C_11_H_15_N_2_O)(C_12_H_8_N_2_)]ClO_4_ or [Cu(*L*)(phen)](ClO_4_) {where *L* refers to the deprotonated form of 2-[(2-di­methyl­amino­ethyl­imino)­meth­yl]phenol} and phen is 1,10-phenanthroline) is a mononuclear mixed ligand copper(II) complex. The Cu^II^ atom is coordinated by two N and one O atoms of the tridentate Schiff base ligand (H*L*) and two N atoms of the 1,10-phenanthroline ligand, resulting in a five-coordinate complex. The asymmetric unit of the title complex contains two crystallographically independent complex cations (**a** and **b**) with a slightly different geometry around the Cu^II^ ion. The value of the trigonality index τ, indicates that in both cations **a** and **b**, the Cu^II^ atoms display a square-pyramidal distorted trigonal–bipyramidal (SPDTBP) geometry, although the distortion is greater for cation **a**.

## Abstract 2

The asymmetric unit of the [Cu(*L*)(phen)](ClO_4_) complex {*L*= 2-[(2-di­methyl­amino­ethyl­imino)­meth­yl]phenol} and phen = 1,10-phenanthroline) contains two crystallographically independent mol­ecules. It consists of alternating layers of he two types of mol­ecules, which stack along the *c* axis.

## 1. Chemical context

The design and synthesis of mixed ligand copper(II) complexes have received much attention as they exhibit promising anti­cancer and nuclease activities compared to simple 1:1 complexes. Palaniandavar and co-workers (Sharma *et al.*, 2020 ▸; Rajendiran *et al.*, 2007 ▸; Selvakumar *et al.*, 2006 ▸) and Chakravarty and co-workers (Goswami *et al.*, 2012 ▸) have reported the X-ray crystal structures of several mixed ligand copper(II) complexes that have biological activity. Recently, our group has reported a series of mixed ligand copper(II) complexes and their biological applications (Karpagam *et al.*, 2019 ▸, 2022 ▸; Radhakrishnan *et al.*, 2021 ▸). Palaniandavar and co-workers (Jaividhya *et al.*, 2012 ▸) prepared the title complex **I** and investigated its DNA binding, cleavage, and anti­cancer activity. It exhibits good cytotoxicity against MCF7 breast cancer cells with an IC_50_ value of 1.20 ± 0.10 µ*M* and against the ME180 human cervical epidermoid carcinoma cells with an IC_50_ value of 24.6 ± 0.10 µ*M* at 48 h incubation (Jaividhya *et al.*, 2012 ▸). However, the crystal structure of complex **I** was not reported. In this work we report the crystal structure of this mixed ligand copper(II) complex.

## 2. Structural commentary

The title compound **I** is of the type {[Cu(*L*)(phen)](ClO_4_)} {where *L* is the deprotonated form of 2-[(2-di­methyl­amino­ethyl­imino)­meth­yl]phenol and phen is 1,10-phenanthroline} is a mononuclear mixed ligand copper(II) complex. The metal atom is coordinated to the tridentate Schiff base ligand (H*L*) through two N and one O atoms and to two N atoms of the 1,10-phenanthroline ligand, resulting in a five-coordinate complex. 
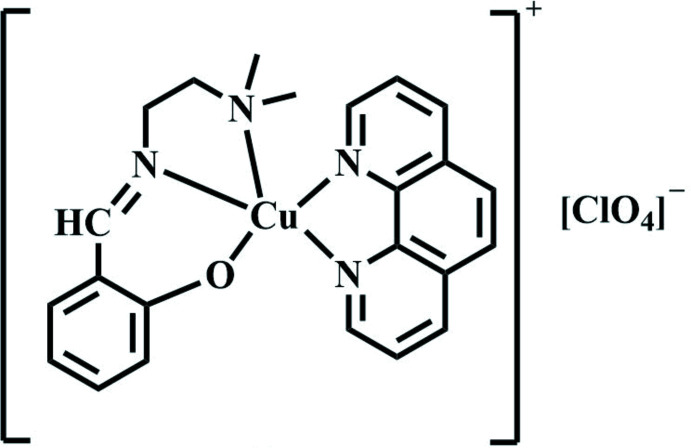


**Figure 1:**
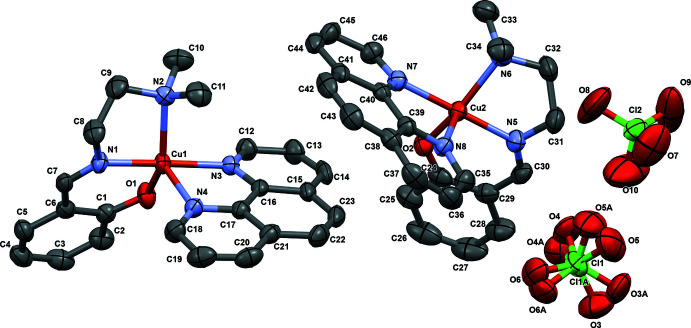
Mol­ecular structures of the crystallographically independent complex cations and the two perchlorate counter-ions with ellipsoids drawn at the 50% probability level; hydrogen atoms have been omitted for clarity.

Complex **I** (Fig. 1 ▸) crystallizes in the ortho­rhom­bic crystal system in the *Pbca* space group. The asymmetric unit contains two crystallographically independent complex cations (**a** and **b**) with a slightly different geometry around the Cu^II^ ion. Selected geometrical parameters are listed in Table 1 ▸. The value of the trigonality index τ suggests that both cations, **a** and **b**, display a square-pyramidal distorted trigonal–bipyramidal (SPDTBP) geometry, with cation **a** being more distorted than cation **b**.

In cation **a**, the Cu1 atom is coordinated by the two nitro­gen atoms (N1, N2) and the phenolate oxygen atom (O1) of the Schiff base primary ligand, and to two nitro­gen (N3, N4) atoms of the phen co-ligand. The value of the trigonality index τ = 0.53 [τ = (β − α)/60, where β = N1—Cu1—N3 = 175.79 (13)° and α = N2—Cu1—O1 = 143.82 (12)°; τ is 0 for a square-pyramidal geometry and 1 for trigonal–bipyramidal] reveals that the coordination environment around Cu1 is best described as having a square-pyramidal distorted trigonal-bipyramidal (SPDTBP) geometry (Addison *et al.*, 1984 ▸; Selvakumar *et al.*, 2006 ▸). The amine nitro­gen atoms (N1, N2) and the phenolate oxygen atom (O1) of the meridionally coordinated Schiff base ligand and one of the imine nitro­gen atoms of phen (N3) occupy the corners of the (Cu1)N_3_O basal plane of this geometry. The other nitro­gen (N4) of the phen ligand occupies the axial position at a distance of 2.251 (3) Å, longer than the equatorial distances [Cu1—O1 = 1.915 (3) Å, Cu1—N1 = 1.923 (3) Å, Cu1—N2 = 2.148 (3) Å, Cu1—N3 = 2.019 (3) Å], which is due to the presence of two electrons in the *d_z_* ^2^ orbital of copper(II). The Cu1—N2_amine_ bond is longer than the Cu1—N1_imine_ bond formed by the Schiff base ligand, which is expected of *sp* ^3^ and *sp* ^2^ hybridizations of the amine (N2) and imine (N1) nitro­gen atoms, respectively. The Cu1—N_imine_ bond distance is shorter than that of *trans* Cu1—N_phen_; this may be attributed to the fact that the azomethine nitro­gen is a stronger base compared with the pyridyl nitro­gen. The bond angles deviate from the ideal trigonal–bipyramidal angles of 90 and 120°, respectively, revealing the presence of significant distortion in the Cu1 coordination geometry.

In cation **b**, the Cu2 ion is coordinated by the two nitro­gen atoms (N5, N6), the phenolate oxygen atom (O2) of the Schiff base primary ligand, and by the two nitro­gen (N7, N8) atoms of the phen co-ligand. As for **a**, cation **b** also exhibits square-pyramidal distorted trigonal–bipyramidal (SPDTBP) geometry (Murphy, Nagle *et al.*, 1997 ▸; Murphy, Murphy *et al.*, 1997 ▸; Nagle *et al.*, 1990 ▸; Rajarajeswari *et al.*, 2014 ▸; Jaividhya *et al.*, 2012 ▸; Radhakrishnan *et al.*, 2021 ▸), but the value of the trigonality index τ is slightly smaller at 0.40 [τ = (β − α)/60, where β = N5—Cu2—N7= 176.38 (14)° and α = O2—Cu2—N6 = 152.71 (12)°], indicating that it is less distorted than cation **a**. Similar to cation **a**, the amine nitro­gen atoms (N5, N6) and the phenolate oxygen atom (O2) of the meridionally coordinated Schiff base ligand and one of the imine nitro­gen atoms of phen occupy the corners of the (Cu2)N_3_O basal plane of this geometry. The other nitro­gen (N8) of the phen ligand occupies the axial position at a distance of 2.238 (3) Å, again longer than the bonds to the equatorial donor atoms [Cu2—O2 = 1.913 (3) Å, Cu2—N5 = 1.919 (3) Å, Cu2—N6 = 2.121 (3) Å, Cu2—N7 = 2.030 (3) Å) but shorter than the axial bond Cu1—N4 of cation **a.** As a result of a slight axial compression of the axial phen nitro­gen in cation **b**, a slight increase of the equatorial phen nitro­gen bond length (Cu2—N7) is observed. On the other hand, the other equatorial bonds in **b** are shorter than in cation **a**. Similar to cation **a**, the Cu2—N6_amine_ bond is longer than the Cu2—N5_imine_ bond formed by the Schiff base ligand, as expected for *sp* ^3^ and *sp* ^2^ hybridizations of the amine (N6) and imine (N5) nitro­gen atoms, respectively. The Cu2—N_imine_ bond distance is shorter than the *trans* Cu2—N_phen_ bond; this is also attributed to stronger basicity of the azomethine nitro­gen compared to the pyridyl nitro­gen. The deviations in the values of the bond angles with respect to the ideal square-pyramidal angles of 90 and 180°, respectively, again reveal a significant distortion in the Cu2 coordination geometry.

## 3. Supra­molecular features

**Figure 2:**
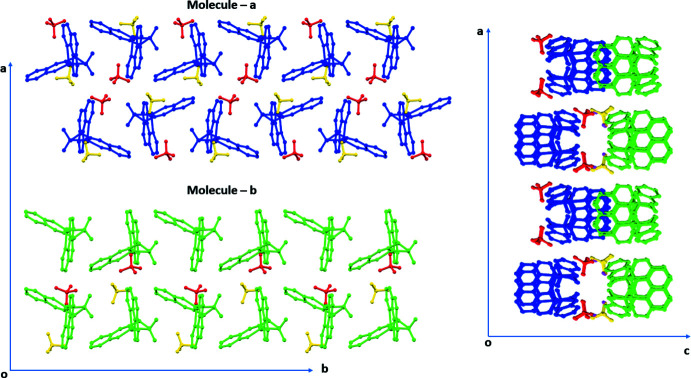
The layered packing arrangement onto the *ab* plane. Complex cations **a** (blue) and **b** (green) are shown on the left side of the figure. The two perchlorate ions are coloured in yellow and red. The relative arrangement of the two layers is shown on the right side of the image.

The two crystallographically independent complex cations stack along the *c*-axis direction with a slightly different packing arrangement. The layered structures formed by complex cations **a** (coloured in blue) and **b** (coloured in green) are shown on the left in Fig. 2 ▸. In this complex, layers parallel to the *ab* plane formed by **a** cations alternate along the *c*-axis with layers of **b** cations. The cations in the supra­molecular structure are linked by weak C—H⋯O hydrogen bonds (Table 2 ▸) mediated by the oxygen atoms of the perchlorate anions. Extensive π–π inter­actions of moderate-to-weak strength are present in the structure, with centroid–centroid distances in the range 3.881 (2) to 4.121 (2) ÅÅ. In addition, C—H⋯π inter­actions (Table 3 ▸) provide enhanced stability to the packing arrangement.

## 4. Database survey

The Cambridge Structural Database (CSD, Version 5.27, updated in November 2022; Groom *et al.*, 2016 ▸) contains no entries with the exact structure of the title compound, [Cu(*L*)(phen)]ClO_4_. However, a few reports are available for similar mixed ligand Cu^II^ complexes containing *L* and di­imine ligands, for example [Cu(*L*)(bpy)]ClO_4_ (Ko *et al.*, 2012 ▸), [Cu(*L*)(dpq)]ClO_4_ and [Cu(*L*)(dmdppz)]ClO_4_ (Jaividhya *et al.*, 2012 ▸) where bpy is 2,2′-bi­pyridine, dpq is dipyrido[3,2-*f*:2′,3′-*h*]quinoxaline and dmdppz = 11,12-di­methyl­dipyrido[3,2-*a*:2′,3′-*c*]phenazine. Similar to the title compound, in these complexes the *N*,*N*,*O*-tridentate Schiff base ligand is coordinated meridionally to the Cu^II^ ion and one of the di­imine nitro­gen atoms is coordinated in an axial position. The value of the trigonality index of the bpy complex (τ = 0.13) is less than for the dpq (τ = 0.37) and dmdppz (τ = 0.39) complexes, as well as the title complex with phen (**a**, τ = 0.53; **b**, τ = 0.40), which exhibits the largest distortion. In addition to these di­imine complexes, there are a few reports on five-coordinate mixed ligand copper(II) complexes bearing *L* and an *N*,*N*-donor ligand such as benzimidazole and an *O*,*O*-donor ligand such as salicyl­aldehyde (Sathya & Murali, 2018 ▸). The *N*,*N*,*O*-tridentate Schiff base ligand is coordinated to the Cu^II^ ion in a meridional fashion and the pyridine nitro­gen of the benzimidazole ligand occupies the axial position, whereas in the salicyl­aldehyde complex, the carbonyl oxygen occupies the axial position. The former complex is distorted from a square-pyramidal geometry and shows a trigonality index τ of 0.25 but the latter complex exhibits only a slight distortion from an ideal square-pyramidal geometry. Similarly, Tadokaro *et al.* (1995 ▸) reported the mol­ecular structure of a mixed ligand complex with *L* and bidentate mono-deprotonated 2,2′-biimidazolate (*N*,*N*-donor) ligands and discussed the existence of a capped-type dimeric hydrogen bond between the mol­ecules. In another case, the authors attempted to synthesize an octa­hedral bis­(*N*-*b*-di­methyl­amino­ethyl­salicyl­adiminato)copper(II) complex (Chieh & Palenik, 1972 ▸). They expected both the tridentate *N*,*N*,*O*-Schiff base ligands to coordinate to the Cu^II^ ion and form an octa­hedral coordination geometry. However, the crystal structure revealed that the Cu^II^ ion is penta­coordinate with one of the di­methyl­amino groups of the ligand not bonded to it. The resulting complex is highly distorted but appears closer to a trigonal–bipyramidal geometry rather than square pyramidal.

## 5. Synthesis and crystallization

The Schiff base-type ligand 2-[(2-di­methyl­amino­ethyl­imino)­meth­yl]phenol (H*L*) was prepared using the synthetic procedure reported by Jaividhya *et al.* (2012 ▸). Complex **I** was prepared by addition of a methano­lic solution (10 mL) of 1,10-phenanthroline (0.1802 g, 1 mmol) and H*L* (0.1949 g, 1 mmol) pretreated with tri­ethyl­amine (139 µL, 1 mmol) to remove the phenolic hydrogen, to a solution of copper(II) perchlorate hexa­hydrate (0.37 g, 1 mmol) in methanol (15 mL) and then stirring at 313 K for 2 h. The green solid obtained was collected by suction filtration, washed with diethyl ether, and then dried under vacuum. A crystal suitable for X-ray diffraction analysis was obtained by dissolving the complex in methanol and allowing it to crystallize.

## 6. Refinement

Crystal data, data collection and structure refinement details are summarized in Table 4 ▸. H atoms were placed in idealized positions and constrained to ride on their parent atoms, with *d*(C—H) = 0.93 Å, *U*_iso_(H) = 1.2*U*_eq_(C) for aromatic, 0.97 Å, *U*_iso_(H) = 1.2*U*_eq_(C) for CH_2_ and 0.96 Å, *U*_iso_(H) = 1.5*U*_eq_(C) for CH_3_ atoms. The hydrogens bound to carbon were refined using standard riding models. The perchlorate ions are disordered. The first, Cl1/O3–O6, was successfully refined with two disorder components which refined to a ratio of 0.611 (15):0.389 (15). Attempts to model the second perchlorate ion (Cl2/O7–O10) did not improve the disagreement factors.

## Supplementary Material

Crystal structure: contains datablock(s) I. DOI: 10.1107/S2056989023001767/dj2061sup1.cif

Structure factors: contains datablock(s) I. DOI: 10.1107/S2056989023001767/dj2061Isup2.hkl

CCDC reference: 2244622

Additional supporting information:  crystallographic information; 3D view; checkCIF report

## Figures and Tables

**Table 1:** Selected geometric parameters (Å, °)

Cu1—O1	1.915 (3)	Cu2—N8	2.238 (3)
Cu1—N1	1.923 (3)	Cl1—O4	1.407 (8)
Cu1—N3	2.019 (3)	Cl1—O5	1.409 (8)
Cu1—N2	2.148 (3)	Cl1—O3	1.415 (8)
Cu1—N4	2.251 (3)	Cl1—O6	1.417 (8)
Cu2—O2	1.913 (3)	Cl2—O9	1.367 (4)
Cu2—N5	1.919 (3)	Cl2—O8	1.373 (4)
Cu2—N7	2.030 (3)	Cl2—O10	1.385 (5)
Cu2—N6	2.121 (3)	Cl2—O7	1.393 (4)
			
O1—Cu1—N1	93.32 (12)	O2—Cu2—N5	93.37 (13)
O1—Cu1—N3	89.55 (12)	O2—Cu2—N7	89.04 (12)
N1—Cu1—N3	175.79 (13)	N5—Cu2—N7	176.36 (14)
O1—Cu1—N2	143.82 (12)	O2—Cu2—N6	152.70 (12)
N1—Cu1—N2	82.91 (13)	N5—Cu2—N6	84.08 (14)
N3—Cu1—N2	96.57 (12)	N7—Cu2—N6	95.01 (13)
O1—Cu1—N4	114.99 (12)	O2—Cu2—N8	107.27 (12)
N1—Cu1—N4	98.12 (12)	N5—Cu2—N8	98.80 (13)
N3—Cu1—N4	77.86 (12)	N7—Cu2—N8	77.87 (12)
N2—Cu1—N4	101.14 (12)	N6—Cu2—N8	99.97 (12)

**Table 2:** Hydrogen-bond geometry (Å, °)

*D*—H⋯*A*	*D*—H	H⋯*A*	*D*⋯*A*	*D*—H⋯*A*
C4—H4⋯O10^i^	0.93	2.51	3.293 (7)	142
C7—H7⋯O4^ii^	0.93	2.59	3.368 (13)	141
C14—H14⋯O2	0.93	2.33	3.191 (5)	153
C22—H22⋯O3*A* ^iii^	0.93	2.56	3.411 (14)	152
C27—H27⋯O5*A* ^iv^	0.93	2.41	3.296 (13)	158
C31—H31*A*⋯O7	0.97	2.59	3.530 (7)	165
C36—H36⋯O6*A* ^iii^	0.93	2.50	3.143 (16)	127
C43—H43⋯O9^v^	0.93	2.53	3.417 (6)	160

Symmetry codes: (i) 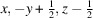 ; (ii) 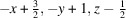 ; (iii) 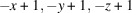 ; (iv) 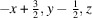 ; (v) 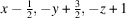 .

**Table 3:** Geometric parameters (Å, °) of C—H⋯π contacts Parameters as defined in *PLATON* (Spek, 2020 ▸). *Cg*1, *Cg*2, *Cg*3 and *Cg*4 are the centroids of the N4/C17–C21, C1–C6, N8/C35–C39 and C24–C29 rings, respectively.

C—H⋯*Cg*	H⋯*Cg*	C⋯*Cg*	C—H⋯*Cg*	Symmetry
C11—H11*B*⋯*Cg*2	2.78	3.447 (5)	128	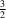 − *x*, 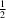 + *y*, *z*
C23—H23⋯*Cg*3	2.80	2.337 (5)	118	*x*, *y*, *z*
C34—H34*C*⋯*Cg*4	2.80	2.434 (6)	124	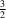 − *x*, 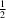 + *y*, *z*
C44—H44⋯*Cg*1	2.78	3.369 (5)	122	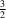 − *x*, 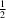 + *y*, *z*

**Table 4:** Experimental details

Crystal data	
Chemical formula	[Cu(C_11_H_15_N_2_O)(C_12_H_8_N_2_)]ClO_4_
*M*_r_	534.44
Crystal system, space group	Orthorhombic, *P* *b* *c* *a*
Temperature (K)	293
*a*, *b*, *c* (Å)	17.8598 (8), 15.0255 (7), 33.920 (2)
*V* (Å^3^)	9102.6 (9)
*Z*	16
Radiation type	Mo *K*α
μ (mm^−1^)	1.12
Crystal size (mm)	0.05 × 0.04 × 0.03

Data collection	
Diffractometer	Xcalibur, Eos, Gemini
Absorption correction	Multi-scan (*CrysAlis PRO*; Agilent, 2013 ▸)
*T*_min_, *T*_max_	0.792, 1.000
No. of measured, independent and observed [*I* > 2σ(*I*)] reflections	28151, 9292, 6329
*R*_int_	0.046
(sin θ/λ)_max_ (Å^−1^)	0.625

Refinement	
*R*[*F* ^2^ > 2σ(*F* ^2^)], *wR*(*F* ^2^), *S*	0.054, 0.130, 1.05
No. of reflections	9292
No. of parameters	663
No. of restraints	154
H-atom treatment	H-atom parameters constrained
Δρ_max_, Δρ_min_ (e Å^−3^)	0.94, −0.42

Computer programs: *CrysAlis PRO* (Agilent, 2013 ▸), *SHELXT2014/5* (Sheldrick, 2015*a* ▸), *SHELXL2018/3* (Sheldrick, 2015*b* ▸), *ORTEPIII* (Burnett & Johnson, 1996 ▸), *Mercury* (Macrae *et al.*, 2020 ▸), *OLEX2* (Dolomanov *et al.*, 2009), *enCIFer* (Allen *et al.*, 2004), *publCIF* (Westrip, 2012) and *PLATON* (Spek, 2020 ▸).

## supplementary crystallographic information

### Crystal data

**Table:**

[Cu(C_11_H_15_N_2_O)(C_12_H_8_N_2_)]ClO_4_	*D*_x_ = 1.560 Mg m^−^^3^
*M**_r_* = 534.44	Mo *K*α radiation, λ = 0.71073 Å
Orthorhombic, *P**b**c**a*	Cell parameters from 28155 reflections
*a* = 17.8598 (8) Å	θ = 3.2–26.4°
*b* = 15.0255 (7) Å	µ = 1.12 mm^−^^1^
*c* = 33.920 (2) Å	*T* = 293 K
*V* = 9102.6 (9) Å^3^	Needle, green
*Z* = 16	0.05 × 0.04 × 0.03 mm
*F*(000) = 4400	

### Data collection

**Table:**

Xcalibur, Eos, Gemini diffractometer	9292 independent reflections
Radiation source: Enhance (Mo) X-ray Source	6329 reflections with *I* > 2σ(*I*)
Graphite monochromator	*R*_int_ = 0.046
Detector resolution: 8.0640 pixels mm^-1^	θ_max_ = 26.4°, θ_min_ = 3.2°
ω scans	*h* = −22→20
Absorption correction: multi-scan (CrysAlisPro; Agilent, 2013)	*k* = −17→18
*T*_min_ = 0.792, *T*_max_ = 1.000	*l* = −40→42
28151 measured reflections	

### Refinement

**Table:**

Refinement on *F*^2^	Primary atom site location: dual
Least-squares matrix: full	Secondary atom site location: difference Fourier map
*R*[*F*^2^ > 2σ(*F*^2^)] = 0.054	Hydrogen site location: inferred from neighbouring sites
*wR*(*F*^2^) = 0.130	H-atom parameters constrained
*S* = 1.05	*w* = 1/[σ^2^(*F*_o_^2^) + (0.0401*P*)^2^ + 10.7563*P*] where *P* = (*F*_o_^2^ + 2*F*_c_^2^)/3
9292 reflections	(Δ/σ)_max_ = 0.001
663 parameters	Δρ_max_ = 0.94 e Å^−^^3^
154 restraints	Δρ_min_ = −0.42 e Å^−^^3^

### Special details

**Table:**

Geometry. All esds (except the esd in the dihedral angle between two l.s. planes) are estimated using the full covariance matrix. The cell esds are taken into account individually in the estimation of esds in distances, angles and torsion angles; correlations between esds in cell parameters are only used when they are defined by crystal symmetry. An approximate (isotropic) treatment of cell esds is used for estimating esds involving l.s. planes.
Refinement. Data collection: A crystal of complex I was mounted on a glass fiber. Data were collected on an Oxford Diffraction Xcalibur EOS Gemini Diffractometer at ambient temperature using graphite-monochromated Mo Kα radiation (λ = 0.7107 Å). The structure was solved with SHELXT (Sheldrick, 2015a) and refined with SHELXL (Sheldrick, 2015b). The graphic interface package PLATON (Spek, 2020), ORTEP (Burnett & Johnson, 1996) and Mercury (Macrae *et al.*, 2020) were used for analysis and generation of images. Non-hydrogen atoms were refined anisotropically.

### Fractional atomic coordinates and isotropic or equivalent isotropic displacement parameters (Å^2^)

**Table:**

	*x*	*y*	*z*	*U*_iso_*/*U*_eq_	Occ. (<1)
Cu1	0.81742 (3)	0.43972 (3)	0.24313 (2)	0.03202 (13)	
Cu2	0.83369 (3)	0.67803 (3)	0.47565 (2)	0.03479 (14)	
O1	0.87548 (16)	0.33345 (18)	0.24931 (8)	0.0417 (7)	
O2	0.88228 (16)	0.56677 (18)	0.46583 (8)	0.0428 (7)	
N1	0.80139 (17)	0.4198 (2)	0.18778 (9)	0.0329 (7)	
N2	0.82238 (17)	0.5766 (2)	0.22508 (9)	0.0340 (7)	
N3	0.82613 (17)	0.4625 (2)	0.30162 (9)	0.0320 (7)	
N4	0.69757 (17)	0.4291 (2)	0.26310 (9)	0.0354 (7)	
N5	0.8188 (2)	0.6527 (2)	0.53054 (10)	0.0431 (9)	
N6	0.83297 (17)	0.8127 (2)	0.49442 (10)	0.0382 (8)	
N7	0.84254 (17)	0.7071 (2)	0.41738 (9)	0.0345 (7)	
N8	0.71511 (18)	0.6647 (2)	0.45510 (9)	0.0363 (8)	
C1	0.8861 (2)	0.2715 (2)	0.22294 (11)	0.0337 (9)	
C2	0.9272 (2)	0.1952 (3)	0.23366 (14)	0.0442 (10)	
H2	0.947412	0.191565	0.258877	0.053*	
C3	0.9382 (2)	0.1260 (3)	0.20769 (15)	0.0482 (12)	
H3	0.964274	0.075734	0.215921	0.058*	
C4	0.9110 (2)	0.1301 (3)	0.16951 (14)	0.0486 (11)	
H4	0.918553	0.083030	0.152142	0.058*	
C5	0.8730 (2)	0.2043 (3)	0.15776 (13)	0.0430 (10)	
H5	0.855520	0.207798	0.131978	0.052*	
C6	0.8596 (2)	0.2757 (2)	0.18367 (11)	0.0337 (9)	
C7	0.8202 (2)	0.3509 (3)	0.16796 (11)	0.0330 (9)	
H7	0.807316	0.349462	0.141397	0.040*	
C8	0.7694 (3)	0.4979 (3)	0.16863 (12)	0.0446 (11)	
H8A	0.717826	0.506449	0.176737	0.054*	
H8B	0.770938	0.491494	0.140189	0.054*	
C9	0.8169 (3)	0.5746 (3)	0.18160 (13)	0.0501 (12)	
H9A	0.866550	0.569102	0.170287	0.060*	
H9B	0.795080	0.629873	0.172247	0.060*	
C10	0.8941 (3)	0.6170 (3)	0.23719 (16)	0.0595 (13)	
H10A	0.934812	0.583264	0.226281	0.089*	
H10B	0.897643	0.616765	0.265432	0.089*	
H10C	0.896568	0.677117	0.227737	0.089*	
C11	0.7615 (3)	0.6296 (3)	0.24258 (14)	0.0537 (12)	
H11A	0.763968	0.625587	0.270795	0.081*	
H11B	0.714111	0.607164	0.233643	0.081*	
H11C	0.766675	0.690688	0.234707	0.081*	
C12	0.8900 (2)	0.4684 (3)	0.32089 (12)	0.0414 (10)	
H12	0.934521	0.462017	0.306958	0.050*	
C13	0.8933 (3)	0.4838 (3)	0.36151 (13)	0.0490 (11)	
H13	0.939374	0.488978	0.374070	0.059*	
C14	0.8291 (3)	0.4912 (3)	0.38247 (12)	0.0465 (11)	
H14	0.830755	0.502418	0.409422	0.056*	
C15	0.7599 (2)	0.4819 (2)	0.36316 (11)	0.0368 (9)	
C16	0.7609 (2)	0.4667 (2)	0.32245 (11)	0.0301 (8)	
C17	0.6921 (2)	0.4504 (2)	0.30172 (11)	0.0329 (9)	
C18	0.6353 (2)	0.4067 (3)	0.24435 (12)	0.0427 (10)	
H18	0.638294	0.391219	0.217854	0.051*	
C19	0.5647 (2)	0.4055 (3)	0.26280 (15)	0.0528 (12)	
H19	0.522211	0.388547	0.248859	0.063*	
C20	0.5598 (2)	0.4294 (3)	0.30130 (15)	0.0495 (11)	
H20	0.513344	0.430513	0.313678	0.059*	
C21	0.6242 (2)	0.4524 (3)	0.32249 (12)	0.0395 (10)	
C22	0.6243 (3)	0.4718 (3)	0.36356 (13)	0.0477 (11)	
H22	0.579133	0.475757	0.377102	0.057*	
C23	0.6889 (3)	0.4844 (3)	0.38292 (13)	0.0471 (11)	
H23	0.687706	0.494976	0.409920	0.057*	
C24	0.8926 (2)	0.5020 (3)	0.49110 (13)	0.0417 (10)	
C25	0.9310 (3)	0.4249 (3)	0.47817 (15)	0.0526 (12)	
H25	0.949904	0.422418	0.452650	0.063*	
C26	0.9405 (3)	0.3534 (3)	0.5032 (2)	0.0691 (16)	
H26	0.964649	0.302654	0.493954	0.083*	
C27	0.9149 (3)	0.3554 (4)	0.5416 (2)	0.0746 (18)	
H27	0.921524	0.306329	0.557955	0.090*	
C28	0.8802 (3)	0.4293 (3)	0.55532 (15)	0.0618 (14)	
H28	0.863768	0.430882	0.581323	0.074*	
C29	0.8686 (2)	0.5037 (3)	0.53095 (13)	0.0440 (11)	
C30	0.8346 (2)	0.5799 (3)	0.54829 (12)	0.0450 (11)	
H30	0.822847	0.576548	0.574952	0.054*	
C31	0.7896 (3)	0.7301 (3)	0.55210 (13)	0.0547 (12)	
H31A	0.795714	0.722062	0.580293	0.066*	
H31B	0.736946	0.738936	0.546402	0.066*	
C32	0.8350 (3)	0.8079 (3)	0.53790 (13)	0.0564 (13)	
H32A	0.814990	0.862515	0.548954	0.068*	
H32B	0.886390	0.801664	0.546723	0.068*	
C33	0.8999 (3)	0.8603 (3)	0.47925 (15)	0.0572 (13)	
H33A	0.944135	0.826873	0.485509	0.086*	
H33B	0.902915	0.918063	0.491273	0.086*	
H33C	0.895941	0.866840	0.451177	0.086*	
C34	0.7662 (3)	0.8622 (3)	0.48093 (14)	0.0536 (12)	
H34A	0.764277	0.861685	0.452652	0.080*	
H34B	0.768947	0.922572	0.490101	0.080*	
H34C	0.721893	0.834536	0.491295	0.080*	
C35	0.6536 (2)	0.6372 (3)	0.47315 (13)	0.0450 (10)	
H35	0.657234	0.618632	0.499238	0.054*	
C36	0.5840 (2)	0.6347 (3)	0.45499 (15)	0.0510 (12)	
H36	0.542601	0.612444	0.468375	0.061*	
C37	0.5769 (2)	0.6653 (3)	0.41728 (15)	0.0513 (12)	
H37	0.530197	0.666457	0.405194	0.062*	
C38	0.6404 (2)	0.6951 (3)	0.39685 (13)	0.0407 (10)	
C39	0.7089 (2)	0.6920 (2)	0.41710 (11)	0.0332 (9)	
C40	0.7771 (2)	0.7126 (2)	0.39697 (11)	0.0304 (8)	
C41	0.7759 (2)	0.7336 (2)	0.35643 (11)	0.0362 (9)	
C42	0.7043 (3)	0.7409 (3)	0.33722 (13)	0.0485 (12)	
H42	0.702256	0.757948	0.310885	0.058*	
C43	0.6401 (3)	0.7234 (3)	0.35672 (13)	0.0479 (11)	
H43	0.594667	0.729995	0.343685	0.058*	
C44	0.8442 (3)	0.7446 (3)	0.33738 (12)	0.0431 (10)	
H44	0.845331	0.757961	0.310617	0.052*	
C45	0.9090 (3)	0.7359 (3)	0.35772 (13)	0.0475 (11)	
H45	0.954831	0.742011	0.345003	0.057*	
C46	0.9061 (2)	0.7176 (3)	0.39810 (13)	0.0440 (10)	
H46	0.950814	0.712544	0.411947	0.053*	
Cl1	0.5801 (4)	0.5616 (5)	0.5795 (2)	0.0407 (13)	0.611 (15)
O3	0.5685 (6)	0.4918 (7)	0.6068 (3)	0.090 (3)	0.611 (15)
O4	0.6566 (5)	0.5808 (9)	0.5742 (3)	0.078 (3)	0.611 (15)
O5	0.5446 (5)	0.6364 (7)	0.5961 (4)	0.101 (3)	0.611 (15)
O6	0.5472 (7)	0.5411 (8)	0.5426 (3)	0.074 (3)	0.611 (15)
Cl1A	0.5833 (7)	0.5590 (9)	0.5804 (4)	0.054 (2)	0.389 (15)
O3A	0.5637 (8)	0.5389 (14)	0.6192 (3)	0.084 (4)	0.389 (15)
O4A	0.6590 (8)	0.5378 (11)	0.5739 (6)	0.071 (4)	0.389 (15)
O5A	0.5740 (10)	0.6517 (7)	0.5773 (5)	0.092 (4)	0.389 (15)
O6A	0.5382 (12)	0.5148 (11)	0.5527 (5)	0.079 (4)	0.389 (15)
Cl2	0.92437 (6)	0.67480 (8)	0.64438 (3)	0.0492 (3)	
O7	0.8485 (2)	0.6921 (4)	0.65003 (14)	0.1193 (18)	
O8	0.9511 (3)	0.6909 (4)	0.60710 (12)	0.127 (2)	
O9	0.9674 (2)	0.7083 (4)	0.67422 (14)	0.141 (2)	
O10	0.9281 (4)	0.5837 (3)	0.6502 (2)	0.168 (3)	

### Atomic displacement parameters (Å^2^)

**Table:**

	*U*^11^	*U*^22^	*U*^33^	*U*^12^	*U*^13^	*U*^23^
Cu1	0.0357 (3)	0.0363 (3)	0.0241 (2)	0.0039 (2)	−0.00180 (19)	−0.0029 (2)
Cu2	0.0392 (3)	0.0375 (3)	0.0277 (3)	−0.0001 (2)	−0.0040 (2)	0.0033 (2)
O1	0.0471 (17)	0.0458 (16)	0.0322 (15)	0.0151 (14)	−0.0085 (13)	−0.0061 (13)
O2	0.0531 (18)	0.0402 (16)	0.0352 (16)	0.0081 (14)	−0.0035 (13)	0.0010 (13)
N1	0.0387 (18)	0.0376 (18)	0.0224 (16)	0.0038 (15)	−0.0015 (14)	0.0024 (14)
N2	0.0305 (17)	0.0343 (18)	0.0371 (19)	0.0007 (14)	−0.0021 (14)	−0.0012 (14)
N3	0.0337 (17)	0.0334 (17)	0.0288 (17)	−0.0014 (14)	−0.0011 (14)	0.0000 (14)
N4	0.0340 (18)	0.0419 (19)	0.0303 (18)	−0.0046 (15)	0.0016 (14)	0.0001 (15)
N5	0.055 (2)	0.044 (2)	0.0304 (19)	−0.0036 (17)	−0.0049 (16)	0.0001 (16)
N6	0.0331 (18)	0.0407 (19)	0.041 (2)	−0.0023 (16)	−0.0041 (15)	−0.0014 (16)
N7	0.0359 (18)	0.0358 (18)	0.0319 (18)	0.0018 (15)	−0.0020 (14)	0.0013 (15)
N8	0.0356 (18)	0.0420 (19)	0.0313 (18)	−0.0037 (15)	0.0006 (15)	0.0022 (15)
C1	0.028 (2)	0.037 (2)	0.036 (2)	0.0013 (17)	0.0017 (17)	0.0009 (18)
C2	0.038 (2)	0.042 (2)	0.052 (3)	0.0057 (19)	−0.005 (2)	0.004 (2)
C3	0.035 (2)	0.032 (2)	0.078 (4)	0.0079 (19)	0.001 (2)	0.000 (2)
C4	0.048 (3)	0.039 (2)	0.059 (3)	−0.002 (2)	0.007 (2)	−0.013 (2)
C5	0.046 (2)	0.042 (2)	0.041 (2)	−0.001 (2)	0.002 (2)	−0.0111 (19)
C6	0.031 (2)	0.033 (2)	0.038 (2)	−0.0021 (17)	0.0046 (17)	−0.0028 (17)
C7	0.034 (2)	0.040 (2)	0.025 (2)	−0.0055 (18)	−0.0011 (16)	−0.0026 (17)
C8	0.052 (3)	0.045 (2)	0.037 (2)	0.013 (2)	−0.010 (2)	0.0051 (19)
C9	0.066 (3)	0.046 (3)	0.039 (3)	0.007 (2)	0.000 (2)	0.011 (2)
C10	0.049 (3)	0.052 (3)	0.077 (4)	−0.008 (2)	−0.005 (3)	0.009 (3)
C11	0.055 (3)	0.041 (2)	0.066 (3)	0.013 (2)	0.002 (2)	−0.002 (2)
C12	0.039 (2)	0.045 (2)	0.039 (2)	−0.001 (2)	−0.0029 (19)	−0.0027 (19)
C13	0.054 (3)	0.055 (3)	0.037 (2)	−0.004 (2)	−0.011 (2)	−0.002 (2)
C14	0.076 (3)	0.039 (2)	0.025 (2)	0.000 (2)	−0.012 (2)	−0.0084 (18)
C15	0.056 (3)	0.028 (2)	0.027 (2)	0.0008 (19)	0.0047 (19)	−0.0028 (16)
C16	0.040 (2)	0.0236 (18)	0.027 (2)	0.0008 (16)	0.0028 (17)	0.0014 (15)
C17	0.037 (2)	0.029 (2)	0.033 (2)	0.0017 (17)	0.0038 (17)	0.0013 (16)
C18	0.039 (2)	0.053 (3)	0.036 (2)	−0.004 (2)	−0.0068 (19)	0.003 (2)
C19	0.040 (3)	0.055 (3)	0.063 (3)	−0.008 (2)	−0.011 (2)	0.018 (2)
C20	0.033 (2)	0.049 (3)	0.066 (3)	0.001 (2)	0.008 (2)	0.016 (2)
C21	0.041 (2)	0.035 (2)	0.042 (2)	0.0032 (19)	0.0109 (19)	0.0041 (18)
C22	0.052 (3)	0.044 (3)	0.047 (3)	0.005 (2)	0.022 (2)	0.000 (2)
C23	0.077 (3)	0.033 (2)	0.031 (2)	0.004 (2)	0.015 (2)	−0.0030 (18)
C24	0.038 (2)	0.037 (2)	0.051 (3)	−0.0039 (19)	−0.017 (2)	−0.001 (2)
C25	0.049 (3)	0.045 (3)	0.063 (3)	0.002 (2)	−0.016 (2)	−0.003 (2)
C26	0.058 (3)	0.040 (3)	0.109 (5)	0.003 (2)	−0.035 (3)	0.003 (3)
C27	0.077 (4)	0.053 (3)	0.094 (5)	−0.009 (3)	−0.040 (4)	0.028 (3)
C28	0.070 (3)	0.057 (3)	0.059 (3)	−0.010 (3)	−0.024 (3)	0.024 (3)
C29	0.044 (2)	0.045 (2)	0.043 (3)	−0.011 (2)	−0.019 (2)	0.009 (2)
C30	0.050 (3)	0.056 (3)	0.029 (2)	−0.014 (2)	−0.0082 (19)	0.010 (2)
C31	0.071 (3)	0.062 (3)	0.031 (2)	0.003 (3)	0.006 (2)	−0.003 (2)
C32	0.080 (4)	0.051 (3)	0.038 (3)	−0.003 (3)	−0.005 (2)	−0.013 (2)
C33	0.055 (3)	0.047 (3)	0.070 (3)	−0.009 (2)	0.004 (3)	−0.006 (2)
C34	0.054 (3)	0.042 (2)	0.065 (3)	0.009 (2)	0.001 (2)	0.002 (2)
C35	0.044 (3)	0.046 (2)	0.045 (3)	0.001 (2)	0.010 (2)	−0.003 (2)
C36	0.037 (2)	0.053 (3)	0.063 (3)	−0.005 (2)	0.009 (2)	−0.012 (2)
C37	0.031 (2)	0.053 (3)	0.070 (3)	0.006 (2)	−0.007 (2)	−0.018 (2)
C38	0.039 (2)	0.036 (2)	0.046 (3)	0.0068 (19)	−0.0100 (19)	−0.0081 (19)
C39	0.037 (2)	0.029 (2)	0.034 (2)	0.0042 (17)	−0.0028 (17)	−0.0019 (17)
C40	0.037 (2)	0.0246 (18)	0.030 (2)	0.0041 (16)	−0.0060 (17)	−0.0011 (16)
C41	0.054 (3)	0.0238 (19)	0.030 (2)	0.0039 (19)	−0.0037 (19)	−0.0013 (16)
C42	0.074 (3)	0.038 (2)	0.034 (2)	0.012 (2)	−0.021 (2)	0.0017 (19)
C43	0.048 (3)	0.048 (3)	0.048 (3)	0.012 (2)	−0.021 (2)	−0.004 (2)
C44	0.066 (3)	0.033 (2)	0.031 (2)	0.003 (2)	0.006 (2)	0.0029 (18)
C45	0.053 (3)	0.046 (3)	0.043 (3)	0.004 (2)	0.020 (2)	0.004 (2)
C46	0.038 (2)	0.047 (3)	0.047 (3)	0.001 (2)	0.002 (2)	0.002 (2)
Cl1	0.037 (2)	0.049 (2)	0.036 (2)	−0.003 (2)	0.0024 (19)	0.005 (2)
O3	0.097 (5)	0.086 (6)	0.087 (6)	−0.013 (5)	−0.011 (5)	0.037 (5)
O4	0.049 (4)	0.125 (7)	0.059 (4)	−0.028 (5)	0.005 (3)	0.003 (6)
O5	0.098 (6)	0.093 (5)	0.111 (6)	0.003 (5)	0.027 (5)	−0.037 (5)
O6	0.072 (5)	0.100 (7)	0.051 (4)	−0.018 (5)	−0.020 (4)	0.003 (4)
Cl1A	0.052 (4)	0.064 (4)	0.046 (4)	−0.009 (4)	−0.017 (3)	−0.006 (4)
O3A	0.078 (6)	0.124 (9)	0.049 (6)	−0.021 (7)	0.019 (5)	0.007 (6)
O4A	0.048 (6)	0.085 (8)	0.078 (7)	0.006 (6)	−0.003 (5)	−0.002 (8)
O5A	0.113 (8)	0.054 (6)	0.109 (8)	0.003 (6)	0.009 (7)	0.004 (6)
O6A	0.088 (7)	0.077 (8)	0.070 (8)	−0.027 (6)	−0.020 (7)	−0.010 (6)
Cl2	0.0434 (6)	0.0572 (7)	0.0469 (6)	−0.0083 (5)	0.0026 (5)	0.0063 (5)
O7	0.050 (2)	0.205 (6)	0.103 (4)	0.011 (3)	0.000 (2)	0.023 (4)
O8	0.106 (3)	0.213 (6)	0.062 (3)	−0.061 (4)	0.011 (2)	0.034 (3)
O9	0.076 (3)	0.261 (7)	0.085 (3)	−0.056 (4)	0.011 (3)	−0.061 (4)
O10	0.222 (7)	0.074 (3)	0.207 (7)	0.021 (4)	0.078 (5)	0.029 (4)

### Geometric parameters (Å, º)

**Table:**

Cu1—O1	1.915 (3)	C18—H18	0.9300
Cu1—N1	1.923 (3)	C19—C20	1.358 (6)
Cu1—N3	2.019 (3)	C19—H19	0.9300
Cu1—N2	2.148 (3)	C20—C21	1.400 (6)
Cu1—N4	2.251 (3)	C20—H20	0.9300
Cu2—O2	1.913 (3)	C21—C22	1.423 (6)
Cu2—N5	1.919 (3)	C22—C23	1.342 (6)
Cu2—N7	2.030 (3)	C22—H22	0.9300
Cu2—N6	2.121 (3)	C23—H23	0.9300
Cu2—N8	2.238 (3)	C24—C25	1.416 (6)
O1—C1	1.305 (4)	C24—C29	1.418 (6)
O2—C24	1.310 (5)	C25—C26	1.379 (7)
N1—C7	1.280 (5)	C25—H25	0.9300
N1—C8	1.457 (5)	C26—C27	1.382 (8)
N2—C11	1.473 (5)	C26—H26	0.9300
N2—C10	1.476 (5)	C27—C28	1.354 (8)
N2—C9	1.479 (5)	C27—H27	0.9300
N3—C12	1.317 (5)	C28—C29	1.406 (6)
N3—C16	1.365 (5)	C28—H28	0.9300
N4—C18	1.325 (5)	C29—C30	1.423 (6)
N4—C17	1.352 (5)	C30—H30	0.9300
N5—C30	1.280 (5)	C31—C32	1.502 (6)
N5—C31	1.468 (5)	C31—H31A	0.9700
N6—C32	1.477 (5)	C31—H31B	0.9700
N6—C34	1.479 (5)	C32—H32A	0.9700
N6—C33	1.485 (5)	C32—H32B	0.9700
N7—C46	1.319 (5)	C33—H33A	0.9600
N7—C40	1.361 (5)	C33—H33B	0.9600
N8—C35	1.324 (5)	C33—H33C	0.9600
N8—C39	1.357 (5)	C34—H34A	0.9600
C1—C2	1.408 (5)	C34—H34B	0.9600
C1—C6	1.415 (5)	C34—H34C	0.9600
C2—C3	1.378 (6)	C35—C36	1.388 (6)
C2—H2	0.9300	C35—H35	0.9300
C3—C4	1.384 (6)	C36—C37	1.365 (6)
C3—H3	0.9300	C36—H36	0.9300
C4—C5	1.365 (6)	C37—C38	1.402 (6)
C4—H4	0.9300	C37—H37	0.9300
C5—C6	1.407 (5)	C38—C39	1.403 (5)
C5—H5	0.9300	C38—C43	1.426 (6)
C6—C7	1.434 (5)	C39—C40	1.431 (5)
C7—H7	0.9300	C40—C41	1.411 (5)
C8—C9	1.497 (6)	C41—C44	1.389 (6)
C8—H8A	0.9700	C41—C42	1.440 (6)
C8—H8B	0.9700	C42—C43	1.349 (6)
C9—H9A	0.9700	C42—H42	0.9300
C9—H9B	0.9700	C43—H43	0.9300
C10—H10A	0.9600	C44—C45	1.354 (6)
C10—H10B	0.9600	C44—H44	0.9300
C10—H10C	0.9600	C45—C46	1.398 (6)
C11—H11A	0.9600	C45—H45	0.9300
C11—H11B	0.9600	C46—H46	0.9300
C11—H11C	0.9600	Cl1—O4	1.407 (8)
C12—C13	1.399 (6)	Cl1—O5	1.409 (8)
C12—H12	0.9300	Cl1—O3	1.415 (8)
C13—C14	1.354 (6)	Cl1—O6	1.417 (8)
C13—H13	0.9300	Cl1A—O3A	1.396 (12)
C14—C15	1.406 (6)	Cl1A—O6A	1.403 (12)
C14—H14	0.9300	Cl1A—O4A	1.406 (12)
C15—C16	1.400 (5)	Cl1A—O5A	1.408 (12)
C15—C23	1.434 (6)	Cl2—O9	1.367 (4)
C16—C17	1.436 (5)	Cl2—O8	1.373 (4)
C17—C21	1.403 (5)	Cl2—O10	1.385 (5)
C18—C19	1.407 (6)	Cl2—O7	1.393 (4)
			
O1—Cu1—N1	93.32 (12)	N4—C18—H18	118.6
O1—Cu1—N3	89.55 (12)	C19—C18—H18	118.6
N1—Cu1—N3	175.79 (13)	C20—C19—C18	118.8 (4)
O1—Cu1—N2	143.82 (12)	C20—C19—H19	120.6
N1—Cu1—N2	82.91 (13)	C18—C19—H19	120.6
N3—Cu1—N2	96.57 (12)	C19—C20—C21	120.4 (4)
O1—Cu1—N4	114.99 (12)	C19—C20—H20	119.8
N1—Cu1—N4	98.12 (12)	C21—C20—H20	119.8
N3—Cu1—N4	77.86 (12)	C20—C21—C17	116.6 (4)
N2—Cu1—N4	101.14 (12)	C20—C21—C22	123.7 (4)
O2—Cu2—N5	93.37 (13)	C17—C21—C22	119.7 (4)
O2—Cu2—N7	89.04 (12)	C23—C22—C21	120.6 (4)
N5—Cu2—N7	176.36 (14)	C23—C22—H22	119.7
O2—Cu2—N6	152.70 (12)	C21—C22—H22	119.7
N5—Cu2—N6	84.08 (14)	C22—C23—C15	121.9 (4)
N7—Cu2—N6	95.01 (13)	C22—C23—H23	119.1
O2—Cu2—N8	107.27 (12)	C15—C23—H23	119.1
N5—Cu2—N8	98.80 (13)	O2—C24—C25	118.2 (4)
N7—Cu2—N8	77.87 (12)	O2—C24—C29	124.6 (4)
N6—Cu2—N8	99.97 (12)	C25—C24—C29	117.2 (4)
C1—O1—Cu1	126.8 (2)	C26—C25—C24	120.4 (5)
C24—O2—Cu2	126.8 (3)	C26—C25—H25	119.8
C7—N1—C8	121.3 (3)	C24—C25—H25	119.8
C7—N1—Cu1	126.9 (3)	C25—C26—C27	121.5 (5)
C8—N1—Cu1	111.6 (2)	C25—C26—H26	119.2
C11—N2—C10	107.9 (3)	C27—C26—H26	119.2
C11—N2—C9	111.3 (3)	C28—C27—C26	119.6 (5)
C10—N2—C9	110.1 (3)	C28—C27—H27	120.2
C11—N2—Cu1	111.9 (2)	C26—C27—H27	120.2
C10—N2—Cu1	110.5 (3)	C27—C28—C29	121.2 (5)
C9—N2—Cu1	105.2 (2)	C27—C28—H28	119.4
C12—N3—C16	118.7 (3)	C29—C28—H28	119.4
C12—N3—Cu1	124.4 (3)	C28—C29—C24	120.1 (4)
C16—N3—Cu1	116.8 (2)	C28—C29—C30	117.4 (4)
C18—N4—C17	117.7 (3)	C24—C29—C30	122.5 (4)
C18—N4—Cu1	132.2 (3)	N5—C30—C29	126.0 (4)
C17—N4—Cu1	110.1 (2)	N5—C30—H30	117.0
C30—N5—C31	121.4 (4)	C29—C30—H30	117.0
C30—N5—Cu2	126.5 (3)	N5—C31—C32	105.4 (4)
C31—N5—Cu2	112.1 (3)	N5—C31—H31A	110.7
C32—N6—C34	110.7 (4)	C32—C31—H31A	110.7
C32—N6—C33	110.5 (3)	N5—C31—H31B	110.7
C34—N6—C33	107.4 (3)	C32—C31—H31B	110.7
C32—N6—Cu2	104.7 (3)	H31A—C31—H31B	108.8
C34—N6—Cu2	113.1 (3)	N6—C32—C31	110.2 (4)
C33—N6—Cu2	110.5 (3)	N6—C32—H32A	109.6
C46—N7—C40	118.6 (3)	C31—C32—H32A	109.6
C46—N7—Cu2	125.1 (3)	N6—C32—H32B	109.6
C40—N7—Cu2	116.2 (2)	C31—C32—H32B	109.6
C35—N8—C39	117.7 (4)	H32A—C32—H32B	108.1
C35—N8—Cu2	132.0 (3)	N6—C33—H33A	109.5
C39—N8—Cu2	110.3 (2)	N6—C33—H33B	109.5
O1—C1—C2	118.6 (4)	H33A—C33—H33B	109.5
O1—C1—C6	124.3 (3)	N6—C33—H33C	109.5
C2—C1—C6	117.0 (4)	H33A—C33—H33C	109.5
C3—C2—C1	121.6 (4)	H33B—C33—H33C	109.5
C3—C2—H2	119.2	N6—C34—H34A	109.5
C1—C2—H2	119.2	N6—C34—H34B	109.5
C2—C3—C4	121.0 (4)	H34A—C34—H34B	109.5
C2—C3—H3	119.5	N6—C34—H34C	109.5
C4—C3—H3	119.5	H34A—C34—H34C	109.5
C5—C4—C3	118.9 (4)	H34B—C34—H34C	109.5
C5—C4—H4	120.5	N8—C35—C36	123.1 (4)
C3—C4—H4	120.5	N8—C35—H35	118.4
C4—C5—C6	121.7 (4)	C36—C35—H35	118.4
C4—C5—H5	119.2	C37—C36—C35	119.3 (4)
C6—C5—H5	119.2	C37—C36—H36	120.3
C5—C6—C1	119.8 (4)	C35—C36—H36	120.3
C5—C6—C7	116.9 (4)	C36—C37—C38	119.7 (4)
C1—C6—C7	123.4 (3)	C36—C37—H37	120.1
N1—C7—C6	124.8 (3)	C38—C37—H37	120.1
N1—C7—H7	117.6	C37—C38—C39	116.9 (4)
C6—C7—H7	117.6	C37—C38—C43	124.3 (4)
N1—C8—C9	105.5 (3)	C39—C38—C43	118.7 (4)
N1—C8—H8A	110.6	N8—C39—C38	123.1 (4)
C9—C8—H8A	110.6	N8—C39—C40	116.6 (3)
N1—C8—H8B	110.6	C38—C39—C40	120.1 (4)
C9—C8—H8B	110.6	N7—C40—C41	121.5 (4)
H8A—C8—H8B	108.8	N7—C40—C39	118.4 (3)
N2—C9—C8	110.2 (3)	C41—C40—C39	120.0 (3)
N2—C9—H9A	109.6	C44—C41—C40	117.8 (4)
C8—C9—H9A	109.6	C44—C41—C42	124.0 (4)
N2—C9—H9B	109.6	C40—C41—C42	118.1 (4)
C8—C9—H9B	109.6	C43—C42—C41	121.2 (4)
H9A—C9—H9B	108.1	C43—C42—H42	119.4
N2—C10—H10A	109.5	C41—C42—H42	119.4
N2—C10—H10B	109.5	C42—C43—C38	121.5 (4)
H10A—C10—H10B	109.5	C42—C43—H43	119.2
N2—C10—H10C	109.5	C38—C43—H43	119.2
H10A—C10—H10C	109.5	C45—C44—C41	120.1 (4)
H10B—C10—H10C	109.5	C45—C44—H44	120.0
N2—C11—H11A	109.5	C41—C44—H44	120.0
N2—C11—H11B	109.5	C44—C45—C46	119.1 (4)
H11A—C11—H11B	109.5	C44—C45—H45	120.4
N2—C11—H11C	109.5	C46—C45—H45	120.4
H11A—C11—H11C	109.5	N7—C46—C45	122.8 (4)
H11B—C11—H11C	109.5	N7—C46—H46	118.6
N3—C12—C13	122.4 (4)	C45—C46—H46	118.6
N3—C12—H12	118.8	O4—Cl1—O5	109.0 (7)
C13—C12—H12	118.8	O4—Cl1—O3	112.2 (7)
C14—C13—C12	119.7 (4)	O5—Cl1—O3	105.2 (6)
C14—C13—H13	120.2	O4—Cl1—O6	109.6 (7)
C12—C13—H13	120.2	O5—Cl1—O6	109.9 (8)
C13—C14—C15	119.5 (4)	O3—Cl1—O6	110.9 (7)
C13—C14—H14	120.3	O3A—Cl1A—O6A	112.6 (12)
C15—C14—H14	120.3	O3A—Cl1A—O4A	109.9 (13)
C16—C15—C14	117.7 (4)	O6A—Cl1A—O4A	109.9 (13)
C16—C15—C23	118.4 (4)	O3A—Cl1A—O5A	104.8 (11)
C14—C15—C23	123.8 (4)	O6A—Cl1A—O5A	110.5 (12)
N3—C16—C15	121.9 (4)	O4A—Cl1A—O5A	109.0 (11)
N3—C16—C17	118.0 (3)	O9—Cl2—O8	115.0 (3)
C15—C16—C17	120.0 (4)	O9—Cl2—O10	103.4 (4)
N4—C17—C21	123.6 (4)	O8—Cl2—O10	106.7 (4)
N4—C17—C16	116.9 (3)	O9—Cl2—O7	112.1 (3)
C21—C17—C16	119.3 (4)	O8—Cl2—O7	115.6 (3)
N4—C18—C19	122.8 (4)	O10—Cl2—O7	102.3 (4)
			
Cu1—O1—C1—C2	−176.3 (3)	Cu2—O2—C24—C25	−179.2 (3)
Cu1—O1—C1—C6	4.2 (5)	Cu2—O2—C24—C29	0.0 (6)
O1—C1—C2—C3	177.6 (4)	O2—C24—C25—C26	−177.3 (4)
C6—C1—C2—C3	−2.9 (6)	C29—C24—C25—C26	3.4 (6)
C1—C2—C3—C4	2.0 (7)	C24—C25—C26—C27	−1.8 (7)
C2—C3—C4—C5	0.1 (7)	C25—C26—C27—C28	−0.5 (8)
C3—C4—C5—C6	−1.2 (6)	C26—C27—C28—C29	1.0 (8)
C4—C5—C6—C1	0.2 (6)	C27—C28—C29—C24	0.8 (7)
C4—C5—C6—C7	179.0 (4)	C27—C28—C29—C30	−177.3 (4)
O1—C1—C6—C5	−178.8 (4)	O2—C24—C29—C28	177.8 (4)
C2—C1—C6—C5	1.8 (5)	C25—C24—C29—C28	−2.9 (6)
O1—C1—C6—C7	2.5 (6)	O2—C24—C29—C30	−4.1 (6)
C2—C1—C6—C7	−176.9 (3)	C25—C24—C29—C30	175.1 (4)
C8—N1—C7—C6	172.5 (4)	C31—N5—C30—C29	−174.6 (4)
Cu1—N1—C7—C6	−2.2 (6)	Cu2—N5—C30—C29	1.9 (6)
C5—C6—C7—N1	177.6 (4)	C28—C29—C30—N5	−178.8 (4)
C1—C6—C7—N1	−3.7 (6)	C24—C29—C30—N5	3.1 (7)
C7—N1—C8—C9	−126.6 (4)	C30—N5—C31—C32	132.7 (4)
Cu1—N1—C8—C9	48.9 (4)	Cu2—N5—C31—C32	−44.2 (4)
C11—N2—C9—C8	−89.4 (4)	C34—N6—C32—C31	85.7 (4)
C10—N2—C9—C8	151.0 (4)	C33—N6—C32—C31	−155.4 (4)
Cu1—N2—C9—C8	32.0 (4)	Cu2—N6—C32—C31	−36.4 (4)
N1—C8—C9—N2	−52.8 (4)	N5—C31—C32—N6	53.2 (5)
C16—N3—C12—C13	−3.8 (6)	C39—N8—C35—C36	0.3 (6)
Cu1—N3—C12—C13	−179.7 (3)	Cu2—N8—C35—C36	179.3 (3)
N3—C12—C13—C14	1.5 (7)	N8—C35—C36—C37	−2.8 (7)
C12—C13—C14—C15	1.0 (7)	C35—C36—C37—C38	2.8 (7)
C13—C14—C15—C16	−1.1 (6)	C36—C37—C38—C39	−0.4 (6)
C13—C14—C15—C23	176.9 (4)	C36—C37—C38—C43	176.1 (4)
C12—N3—C16—C15	3.6 (5)	C35—N8—C39—C38	2.3 (6)
Cu1—N3—C16—C15	179.8 (3)	Cu2—N8—C39—C38	−177.0 (3)
C12—N3—C16—C17	−173.3 (3)	C35—N8—C39—C40	−173.7 (3)
Cu1—N3—C16—C17	3.0 (4)	Cu2—N8—C39—C40	7.1 (4)
C14—C15—C16—N3	−1.2 (5)	C37—C38—C39—N8	−2.2 (6)
C23—C15—C16—N3	−179.3 (3)	C43—C38—C39—N8	−179.0 (4)
C14—C15—C16—C17	175.6 (3)	C37—C38—C39—C40	173.6 (4)
C23—C15—C16—C17	−2.5 (5)	C43—C38—C39—C40	−3.1 (6)
C18—N4—C17—C21	−1.7 (6)	C46—N7—C40—C41	−3.3 (5)
Cu1—N4—C17—C21	178.2 (3)	Cu2—N7—C40—C41	179.1 (3)
C18—N4—C17—C16	174.6 (3)	C46—N7—C40—C39	173.5 (3)
Cu1—N4—C17—C16	−5.5 (4)	Cu2—N7—C40—C39	−4.1 (4)
N3—C16—C17—N4	2.2 (5)	N8—C39—C40—N7	−2.6 (5)
C15—C16—C17—N4	−174.7 (3)	C38—C39—C40—N7	−178.7 (3)
N3—C16—C17—C21	178.7 (3)	N8—C39—C40—C41	174.2 (3)
C15—C16—C17—C21	1.8 (5)	C38—C39—C40—C41	−1.8 (5)
C17—N4—C18—C19	0.7 (6)	N7—C40—C41—C44	2.9 (5)
Cu1—N4—C18—C19	−179.2 (3)	C39—C40—C41—C44	−173.9 (3)
N4—C18—C19—C20	1.0 (7)	N7—C40—C41—C42	−178.1 (3)
C18—C19—C20—C21	−1.8 (7)	C39—C40—C41—C42	5.2 (5)
C19—C20—C21—C17	0.9 (6)	C44—C41—C42—C43	175.4 (4)
C19—C20—C21—C22	−175.2 (4)	C40—C41—C42—C43	−3.6 (6)
N4—C17—C21—C20	0.9 (6)	C41—C42—C43—C38	−1.4 (6)
C16—C17—C21—C20	−175.3 (3)	C37—C38—C43—C42	−171.7 (4)
N4—C17—C21—C22	177.1 (4)	C39—C38—C43—C42	4.8 (6)
C16—C17—C21—C22	0.9 (6)	C40—C41—C44—C45	−0.5 (6)
C20—C21—C22—C23	173.0 (4)	C42—C41—C44—C45	−179.5 (4)
C17—C21—C22—C23	−2.9 (6)	C41—C44—C45—C46	−1.3 (6)
C21—C22—C23—C15	2.1 (6)	C40—N7—C46—C45	1.4 (6)
C16—C15—C23—C22	0.6 (6)	Cu2—N7—C46—C45	178.7 (3)
C14—C15—C23—C22	−177.4 (4)	C44—C45—C46—N7	0.9 (6)

### Hydrogen-bond geometry (Å, º)

**Table:**

*D*—H···*A*	*D*—H	H···*A*	*D*···*A*	*D*—H···*A*
C4—H4···O10^i^	0.93	2.51	3.293 (7)	142
C7—H7···O4^ii^	0.93	2.59	3.368 (13)	141
C14—H14···O2	0.93	2.33	3.191 (5)	153
C22—H22···O3*A*^iii^	0.93	2.56	3.411 (14)	152
C27—H27···O5*A*^iv^	0.93	2.41	3.296 (13)	158
C31—H31*A*···O7	0.97	2.59	3.530 (7)	165
C36—H36···O6*A*^iii^	0.93	2.50	3.143 (16)	127
C43—H43···O9^v^	0.93	2.53	3.417 (6)	160

Symmetry codes: (i) *x*, −*y*+1/2, *z*−1/2; (ii) −*x*+3/2, −*y*+1, *z*−1/2; (iii) −*x*+1, −*y*+1, −*z*+1; (iv) −*x*+3/2, *y*−1/2, *z*; (v) *x*−1/2, −*y*+3/2, −*z*+1.

## References

[bb1] Addison, A. W., Rao, T. N., Reedijk, J., van Rijn, J. & Verschoor, G. C. (1984). *J. Chem. Soc. Dalton Trans.* pp. 1349–1356.

[bb2] Agilent (2013). *CrysAlis PRO*. Agilent Technologies, Yarnton, England.

[bb3] Burnett, M. N. & Johnson, C. K. (1996). *ORTEPIII*. Report ORNL-6895. Oak Ridge National Laboratory, Tennessee, USA.

[bb4] Chieh, P. C. & Palenik, G. J. (1972). *Inorg. Chem.* **11**, 816–819.

[bb5] Goswami, T. K., Gadadhar, S., Roy, M., Nethaji, M., Karande, A. A. & Chakravarty, A. R. (2012). *Organometallics*, **31**, 3010–3021.

[bb25] Groom, C. R., Bruno, I. J., Lightfoot, M. P. & Ward, S. C. (2016). *Acta Cryst*. B**72**, 171–179. 10.1107/S2052520616003954 PMC4822653 27048719

[bb6] Jaividhya, P., Dhivya, R., Akbarsha, M. A. & Palaniandavar, M. (2012). *J. Inorg. Biochem.* **114**, 94–105. 10.1016/j.jinorgbio.2012.04.018 22841366

[bb7] Karpagam, S., Kartikeyan, R., Paravai Nachiyar, P., Velusamy, M., Kannan, M., Krishnan, M., Chitgupi, U., Lovell, J. F., Abdulkader Akbarsha, M. & Rajendiran, V. (2019). *J. Coord. Chem.* **72**, 3102–3127.

[bb8] Karpagam, S., Mamindla, A., Kumar Sali, V., Niranjana, R. S., Periasamy, V. S., Alshatwi, A. A., Akbarsha, M. A. & Rajendiran, V. (2022). *Inorg. Chim. Acta*, **531**, 120729–120740.

[bb10] Ko, B., Chang, C., Lai, S., Lai, F. & Lin, C. (2012). *Polyhedron*, **45**, 49–54.

[bb11] Macrae, C. F., Sovago, I., Cottrell, S. J., Galek, P. T. A., McCabe, P., Pidcock, E., Platings, M., Shields, G. P., Stevens, J. S., Towler, M. & Wood, P. A. (2020). *J. Appl. Cryst.* **53**, 226–235. 10.1107/S1600576719014092 PMC6998782 32047413

[bb12] Murphy, G., Murphy, C., Murphy, B. & Hathaway, B. (1997). *J. Chem. Soc. Dalton Trans.* pp. 2653–2660.

[bb13] Murphy, G., Nagle, P., Murphy, B. & Hathaway, B. (1997). *J. Chem. Soc. Dalton Trans.* pp. 2645–2652.

[bb14] Nagle, P., O’Sullivan, E., Hathaway, B. J. & Muller, E. (1990). *J. Chem. Soc. Dalton Trans.* pp. 3399–3406.

[bb15] Radhakrishnan, K., Khamrang, T., Sambantham, K., Sali, V. K., Chitgupi, U., Lovell, J. F., Mohammad, A. A. & Venugopal, R. (2021). *Polyhedron*, **194**, 114886–114899.

[bb16] Rajarajeswari, C., Ganeshpandian, M., Palaniandavar, M., Riyasdeen, A. & Akbarsha, M. A. (2014). *J. Inorg. Biochem.* **140**, 255–268. 10.1016/j.jinorgbio.2014.07.016 25199844

[bb17] Rajendiran, V., Karthik, R., Palaniandavar, M., Stoeckli-Evans, H., Periasamy, V. S., Akbarsha, M. A., Srinag, B. S. & Krishnamurthy, H. (2007). *Inorg. Chem.* **46**, 8208–8221. 10.1021/ic700755p 17784750

[bb18] Sathya, V. & Murali, M. (2018). *Inorg. Chem. Commun.* **92**, 55–59.

[bb19] Selvakumar, B., Rajendiran, V., Uma Maheswari, P., Stoeckli-Evans, H. & Palaniandavar, M. (2006). *J. Inorg. Biochem.* **100**, 316–330. 10.1016/j.jinorgbio.2005.11.018 16406550

[bb20] Sharma, M., Ganeshpandian, M., Majumder, M., Tamilarasan, A., Sharma, M., Mukhopadhyay, R., Islam, N. S. & Palaniandavar, M. (2020). *Dalton Trans.* **49**, 8282–8297. 10.1039/d0dt00928h 32510543

[bb21] Sheldrick, G. M. (2015*a*). *Acta Cryst.* A**71**, 3–8.

[bb22] Sheldrick, G. M. (2015*b*). *Acta Cryst.* C**71**, 3–8.

[bb23] Spek, A. L. (2020). *Acta Cryst.* E**76**, 1–11. 10.1107/S2056989019016244 PMC6944088 31921444

[bb24] Tadokoro, M., Toyoda, J., Isobe, K., Itoh, T., Miyazaki, A., Enoki, T. & Nakasuji, K. (1995). *Chem. Lett.* **24**, 613–614.

